# Remedies That Enslave: How to Use Them Rightly

**Published:** 1897-09

**Authors:** 


### REMEDIES THAT ENSLAVE: HOW TO USE THEM RIGHTLY.

It seems hardly necessary to explain what we mean by "remedies that enslave," but to avoid misunderstanding we name the following as representative of the class: Opium, chloral, alcohol, cocaine. We might also add, in a general way, the unintelligent continued use of laxatives.

The baneful effects of the careless use of any of these have been witnessed by every practitioner of a few years' experience —in fact, are seen by nearly all students in college clinics. They not only enslave the patient, ruin the health, dull the moral sensibilities, and degrade their victims into tiresome and worrying dependents, but they disgrace whole families, wreck happy homes, and leave a train of unimaginable misery upon all sides, the victims having no further excuse to live, and yet deferring for a long time the harvest of the undertaker. They are a blot upon our civilization and a disgrace to an honored profession. The man who by his carelessness has been the cause of a drug habit is indeed to be pitied if he is able to realize the injury he has inflicted and the almost utter hopelessness of its remediability.

"Can these terrible results be avoided?" "Can they always be prevented?" are most pertinent questions, and to them may very properly be added this other—"If so, how?" Whilst to say that all are preventable would be too inelastically sweeping a statement, we feel safe in saying that the constant observance of two single rules would make the formation of a habit impossible in nearly every instance.

In the consideration of this, as in that of any other similar subject, it is proper to apply the accepted principle that one of the most essential things to do in the cure of a disease is to remove its cause, and that other one, that diseases should be prevented whenever possible by the avoidance of their cause. The attempt to cure these habits is a worthy effort as well as a profitable, though trying, business; but it so commonly remains merely an attempt, or is only effective temporarily, that prevention is infinitely the better plan. This, then, brings us to the cause of drug habits—why they are formed and at what time.

At least two essential factors produce drug enslavement, and these are predisposition and opportunity. The former is so difficult to estimate that we make no attempt in this limited space to define or describe what little we could about it that might be of practical use, but go at once to the latter, opportunity.

We have elsewhere repeatedly laid down the proposition that the system may safely be tendered those remedies which it needs in sufficient quantity to be effective, and for as long a time as they are required, without any danger of the formation of a habit, provided they are withdrawn when no longer demanded. This rule, broadly interpreted, will, we believe, cover all cases. It is the key to the avoidance of drug habits.

A sad instance came under our notice some sixteen years ago of the alcohol habit formed by an estimable lady through the active insistence of her medical adviser that she continue beyond the period of safety. She was convalescing from a long attack of an acute illness. Reaction was naturally slow. The physician was a liberal drinker, stout, florid, often maudlin, and always positive. He put her upon wines, to which she had never been accustomed and to which she objected. He insisted. She expressed her fears of the ultimate effect, especially in view of the fact that drunkenness existed in her progenitors. He became imperative. She yielded. When convalescence was well advanced she desired to discontinue, but he objected and even increased the allowance! He continued insisting on their use for several months—and she acquiesced. That settled it. She was never able to discontinue. He died before he knew the harm he had wrought. Then she came under our care. She was a constant drinker, intoxicated almost daily. Her child and home were neglected. A loyal husband did his full duty. Eventually she was placed in a home for inebriates and returned "cured," remaining so for a few months and again relapsing. She was never "cured" again, and died one morning when about to rise. Her heart and liver had undergone fatty degeneration. From an estimable young lady of prepossessing figure and appearance, she had in four years degenerated into a senseless, dyspeptic, vixenish, stupid, and bloated, figureless creature, much better dead than alive, a pitiable burden alike to herself and friends.

This is one of many cases within our own personal experience. The offense of transforming a useful human being into such rubbish of undesirableness is infinitely greater and far-reaching in its banefulness than the immediate loss of life upon the operating table or from defective treatment upon the sick bed. The latter may be excusable, because of extenuating circumstances, the former never, because readily avoidable.

The first rule we should have observed is to give these enslaving remedies only when they are indicated, and then give fully, freely and fearlessly. But withdraw them as the need of them lessens, and stop them altogether the moment they are no longer required. This has always been our plan, and we know that no drug habit has ever followed its careful adherence. Our second rule serves only to make the first doubly sure and consists in not letting the patient know what is being used. Nor should anyone about the patient be permitted to know, lest it be injudiciously imparted during some unguarded moment. Patients should be protected against themselves. And the desirability of keeping the patient in ignorance of what is being given is another effective reason in favor of the physician carrying his own drugs.

Nor should these remedies be carelessly resorted to. We have upon numerous occasions used half grain tablets of the extract of nux vomica in the place of alcohol, which was entirely withdrawn. This has been our custom even with habitual heavy drinkers suffering with acute lobar pneumonitis, and the results have been better than could be achieved in the same cases with alcohol, as proven by actual tentative experiments. Thus, also, can many a pain or night of sleeplessness be corrected without the use of opium or of chloral. To neglect a readily discovered cause of pain or insomnia and then helplessly have recourse to opium and chloral is almost tantamount to malpractice—it is, at least, inexcusable.

Avoid, then, the use of enslaving drugs when they are not really necessary and never let a patient know what remedy is being used if it is one of this class. Rigid adherence to these rules will never permit the formation of a drug habit; deviation therefrom is almost sure to cause it.—*Medical Council.*

